# Supplementary material for: Carotid intima-media thickness and risk of atherosclerosis in multiple sclerosis: A cross-sectional study
Source: PLoS One. 2024 Nov 19;19(11):e0314031. doi: 10.1371/journal.pone.0314031 (PMC11575821; doi:10.1371/journal.pone.0314031)
Supplement: S1 File — Each table explores specific variables and findings that support the main results of the study. (DOCX) [file pone.0314031.s001.docx]

**Collinearity Statistics in Linear Regression Model for CIMT in pwMS and control subjects**

Supplementary Table 1 presents the collinearity statistics for predictors included in the linear regression model assessing CIMT among pwMS and control subjects. The tolerance values indicate the degree of multicollinearity, with values ranging from 0.89 to 0.95. Correspondingly, the variance inflation factor (VIF) values range from 1.08 to 1.12. These results suggest that there is minimal multicollinearity among the predictors included in the model, as indicated by tolerance values close to 1 and VIF values close to 1, meeting the assumption of multicollinearity in regression analysis (S1 table).

**S1 Table. Collinearity Statistics in Linear Regression Model for CIMT in pwMS and control subjects**

|  | **MS status** | **Sex** | **Age** | **BMI** |
| --- | --- | --- | --- | --- |
|  |  |  |  |  |
| **Tolerance** | 0.89 | 0.92 | 0.95 | 0.95 |
| **VIF** | 1.12 | 1.08 | 1.04 | 1.05 |

**Abbreviation;** MS: multiple sclerosis. BMI: Body Mass Index, VIF: variance inflation factor

**Normality of Residuals in Linear Regression Model for CIMT in pwMS and control subjects**

The normality of residuals in the linear regression model for CIMT was assessed among pwMS and control subjects. The mean residual value was 0 with a standard error (SE) of -0.007, and the median of -0.003. The skewness of residuals was 0.26±0.157, indicating a slightly right-skewed distribution, while the kurtosis value of 0.58±0.31 suggests a near-normal distribution with minimal tails. The Kolmogorov-Smirnov test statistic was 0.044 with a corresponding p-value of 0.2, indicating no significant deviation from normality. Overall, these results suggest that the residuals in the linear regression model exhibit satisfactory normality, meeting one of the crucial assumptions of the analysis (S2 table).

**S2 Table. Normality of Residuals in Linear Regression Model for CIMT in pwMS and control subjects**

| **Mean** | **SE** | **Median** | **Skewness** | **Kurtosis** | **Kolmogorov-Smirnov** | |
| --- | --- | --- | --- | --- | --- | --- |
|  |  |  |  |  | **Statistic** | **sig** |
| 0 | 0.007 | -0.003 | 0.26±0.157 | 0.58±0.31 | 0.04 | 0.2 |

**Abbreviation**; SE: Standard Error

**Correlations among variables in Linear Regression Model for CIMT in pwMS and control subjects**

The correlation matrix presented in the table demonstrates the relationships among variables included in the Linear Regression Model for CIMT in pwMS and control subjects. The CIMT exhibits a positive Spearman correlation with age (ρ = 0.292, *p* < 0.001) and sex (ρ = 0.205, *p* < 0.001), indicating that higher values of CIMT tend to be associated with older age and male sex. However, the Spearman correlation between CIMT and BMI is relatively weak (ρ = 0.093, *p* = 0.148), suggesting a less significant relationship. Age also shows a positive Spearman correlation with sex (ρ = -0.102, *p* = 0.001), indicating a slight tendency for younger participants to be female. However, age demonstrates a weaker Spearman correlation with BMI (ρ = 0.205, *p* < 0.001) compared to its correlation with CIMT. BMI exhibits a weak positive Spearman correlation with age (ρ = 0.205, *p* < 0.001) and a very weak negative correlation with sex (ρ = -0.056, *p* = 0.601). Furthermore, it is important to note that none of the variables showed strong correlations with each other, indicating independence among the variables in the regression model. This lack of strong correlation suggests that multicollinearity, which can complicate the interpretation of regression analyses, is not a significant issue in this model.

**S3 Table. Correlations among variables in Linear Regression Model for CIMT in pwMS and control subjects**

| **Variable** | **CIMT** | **Age** | **BMI** | **Sex** |
| --- | --- | --- | --- | --- |
| **CIMT** | 1 | 0.292** | 0.093 | 0.205** |
| **Age** | 0.292 | 1 | 0.205** | -0.102 |
| **BMI** | 0.093 | 0.205** | 1 | -0.056 |
| **Sex** | 0.205** | -0.102 | -0.056 | 1 |

**Abbreviation**; CIMT: Carotid Intima-Media Thickness, BMI: Body Mass Index

**: *p*<0.001

**Bonferroni post hoc test for effect of MS disease course on CIMT**

**S4 Table. Bonferroni post hoc test (complete version)**

| **MS course** | | **Mean Difference (I-J)** | **SE** | **Sig.** | **95% CI** |
| --- | --- | --- | --- | --- | --- |
| **RRMS** | **SPMS** | -0.13952^*^ | 0.03129 | 0.000 | (-0.2236, -0.0554) |
|  | **PPMS** | -0.12042 | 0.05867 | 0.255 | (-0.2781, 0.0372) |
|  | **PRMS** | -0.04875 | 0.09923 | 1.000 | (-0.3154, 0.2179) |
| **SPMS** | **PPMS** | 0.01910 | 0.06278 | 1.000 | (-0.1496, 0.1878) |
|  | **PRMS** | 0.09077 | 0.10171 | 1.000 | (-0.1825, 0.3758) |
| **PPMS** | **PRMS** | 0.07167 | 0.11318 | 1.000 | (-0.2324, 0.3758) |

**Abbreviation**; SE: Standard Error, CI: Confidence Interval, RRMS: Relapsing-Remitting Multiple Sclerosis, SPMS: Secondary Progressive Multiple Sclerosis, PRMS: Progressive-Relapsing Multiple Sclerosis, PPMS: Primary-Progressive Multiple Sclerosis, *:*p*<0.001

**Fitness of model for linear regression model examining the predictors of CIMT in pwMS**

In the linear regression model examining the predictors of CIMT in pwMS, age emerged as a significant predictor (β = 0.007, p < 0.001), with a notably small p-value indicative of its strong association with CIMT. Conversely, the association between BMI and CIMT was marginally significant (β = 0.008, *p* = 0.064), suggesting a weaker relationship compared to age. It's noteworthy that the normality of residuals in the model was violated, as indicated by the significant Kolmogorov-Smirnov test (*p* = 0.015). However, the robustness of the estimates was supported by the sample size (n = 114) and the consistency of results across multiple regression diagnostics.

**S5 Table. Normality of Residuals in Stepwise Linear Regression Model for CIMT Predictors in pwMS**

| **Mean** | **SE** | **Median** | **Skewness** | **Kurtosis** | **Kolmogorov-Smirnov** | |
| --- | --- | --- | --- | --- | --- | --- |
|  |  |  |  |  | **Statistic** | **sig** |
| 0 | 0.010 | -0.016 | 0.565±0.226 | 0.302±0.449 | 0.094 | 0.015 |

Abbreviation; SE: Standard Error

**S6 Table. Heteroskedasticity test in Stepwise Linear Regression Model for CIMT Predictors in pwMS**

| **Test** | **Chi square** | **SE** | **sig** |
| --- | --- | --- | --- |
| **Modified Breusch-Pagan**  Abbreviation; SE: Standard Error | 0.936 | 1 | 0.333 |

Collinearity statistics were assessed to evaluate multicollinearity among predictors in the stepwise linear regression model for CIMT in pwMS. Tolerance values ranged from 0.470 to 0.983, and Variance Inflation Factor (VIF) values ranged from 1.017 to 2.128 across different models. These values suggest no significant multicollinearity, indicating that the predictors included in the model were not highly correlated

**S7 Table. Collinearity Statistics in Stepwise Linear Regression Model for CIMT Predictors in pwMS**

|  | | **Duration of disease** | **SPMS** | **PPMS** | **PRMS** | **EDSS** | **BMI** | **Age** |
| --- | --- | --- | --- | --- | --- | --- | --- | --- |
| **Model 1** | **Tolerance** | 0.614 | 0.503 | 0.882 | 0.983 | 0.543 | - | - |
|  | **VIF** | 1.628 | 1.989 | 1.134 | 1.017 | 1.842 | - | - |
| **Model 2** | **Tolerance** | 0.612 | 0.487 | 0.875 | 0.982 | 0.539 | 0.941 | - |
|  | **VIF** | 1.635 | 2.054 | 1.142 | 1.019 | 1.854 | 1.063 | - |
| **Model 3** | **Tolerance** | 0.601 | 0.470 | 0.841 | 0.982 | 0.487 | 0.921 | 0.590 |
|  | **VIF** | 1.665 | 2.128 | 1.189 | 1.019 | 2.055 | 1.085 | 1.695 |

Abbreviation; RRMS: Relapsing-Remitting Multiple Sclerosis, SPMS: Secondary Progressive Multiple Sclerosis, PRMS: Progressive-Relapsing Multiple Sclerosis, PPMS: Primary-Progressive Multiple Sclerosis, EDSS: Expanded Disability Status Scale, BMI: Body Mass Index, VIF: Variance Inflation Factor

.

Correlation analysis, using Spearman's ρ, revealed significant associations among predictors in the stepwise linear regression model for CIMT in pwMS. Notably, RRMS exhibited a strong negative correlation with SPMS (ρ = -0.383, *p* < 0.01). Age demonstrated a strong positive correlation with CIMT (ρ = 0.612, p < 0.01), while EDSS showed a moderate positive correlation with CIMT (ρ = 0.356, *p* < 0.01). Additionally, BMI exhibited a weak positive correlation with CIMT (ρ = 0.323, *p* < 0.01). Duration of disease, PPMS, and PRMS demonstrated varied correlations with CIMT, suggesting diverse impacts of these variables on CIMT in pwMS.

These findings indicate that while there are correlations among predictors, multicollinearity is not severe. However, it's essential to note the strong negative correlation between RRMS and SPMS, which may influence the interpretation of the regression model results. Each predictor continues to contribute uniquely to the variance in CIMT in pwMS

**S8 Table. Correlations among predictors in stepwise Linear Regression Model for CIMT in pwMS**

|  | **CIMT** | **EDSS** | **Age** | **BMI** | **duration of disease** | **RRMS** | **SPMS** | **PPMS** | **PRMS** |
| --- | --- | --- | --- | --- | --- | --- | --- | --- | --- |
| **CIMT** | 1.000 | .356** | 0.612** | 0.323** | 0.194* | -0.383** | 0.342** | 0.125 | 0.029 |
| **EDSS** | 0.356** | 1.000 | 0.585** | 0.087 | 0.484** | -0.605** | 0.586** | 0.136 | 0.001 |
| **Age** | 0.612** | 0.585** | 1.000 | 0.276** | 0.393** | -0.543** | 0.510** | 0.147 | 0.014 |
| **BMI** | 0.323** | 0.087 | 0.276** | 1.000 | 0.154 | -0.215* | 0.212* | 0.028 | 0.024 |
| **Duration of disease** | 0.194* | 0.484** | 0.393** | 0.154 | 1.000 | -0.438** | 0.551** | -0.156 | 0.030 |
| **RRMS** | -0.383** | -0.605** | -0.543** | -0.215* | -0.438** | 1.000 | -0.834** | -0.362** | -0.205* |
| **SPMS** | 0.342** | 0.586** | 0.510** | 0.212* | 0.551** | -0.834** | 1.000 | -0.128 | -0.073 |
| **PPMS** | 0.125 | 0.136 | 0.147 | 0.028 | -0.156 | -0.362** | -0.128 | 1.000 | -0.031 |
| **PRMS** | 0.029 | 0.001 | 0.014 | 0.024 | 0.030 | -0.205* | -0.073 | -0.031 | 1.000 |

Abbreviation; CIMT: Carotid Intima-Media Thickness, EDSS: Expanded Disability Status Scale, BMI: Body Mass Index, RRMS: Relapsing-Remitting Multiple Sclerosis, SPMS: Secondary Progressive Multiple Sclerosis, PRMS: Progressive-Relapsing Multiple Sclerosis, PPMS: Primary-Progressive Multiple Sclerosis.
