# Supplementary material for: Carotid intima-media thickness and risk of atherosclerosis in multiple sclerosis: A cross-sectional study
Source: PLoS One. 2024 Nov 19;19(11):e0314031. doi: 10.1371/journal.pone.0314031 (PMC11575821; doi:10.1371/journal.pone.0314031)
Supplement: Attachment — Submitted filename: review2.docx [file pone.0314031.s004.docx]

Dear authors,

Thank you very much for the time and effort you put into answering each question and correcting the request in accordance with the request of the Editor and reviewers.

I believe that all the answers are satisfactory and I suggest that the manuscript be published.
