# Supplementary material for: Carotid intima-media thickness and risk of atherosclerosis in multiple sclerosis: A cross-sectional study
Source: PLoS One. 2024 Nov 19;19(11):e0314031. doi: 10.1371/journal.pone.0314031 (PMC11575821; doi:10.1371/journal.pone.0314031)
Supplement: Attachment — Submitted filename: To the editor.docx [file pone.0314031.s005.docx]

Dear Editor,

I believe that now the authors have answered all questions and all requests in a satisfactory manner and that the work can be published without further corrections.

Sincerely
